# Cancer-Associated Fibroblasts Differentiated by Exosomes Isolated from Cancer Cells Promote Cancer Cell Invasion

**DOI:** 10.3390/ijms21218153

**Published:** 2020-10-31

**Authors:** Kimin Kim, Yeh Joo Sohn, Ruri Lee, Hye Ju Yoo, Ji Yoon Kang, Nakwon Choi, Dokyun Na, Ju Hun Yeon

**Affiliations:** 1 Department of Integrative Biosciences, University of Brain Education (UBE), Cheonan 31228, Korea; kimini1127@naver.com (K.K.); spielian@naver.com (Y.J.S.); skin09_@naver.com (R.L.); hyeju_yoo@naver.com (H.J.Y.); 2 Center for BioMicrosystems, Brain Science Institute, Korea Institute of Science and Technology (KIST), Seoul 02792, Korea; jykang@kist.re.kr (J.Y.K.); nakwon.choi@kist.re.kr (N.C.); 3 Division of Bio-Medical Science & Technology (Biomedical Engineering), KIST School, Korea University of Science and Technology (UST), Seoul 02792, Korea; 4 Department of Biomedical Engineering, Chung-Ang University, Seoul 06974, Korea

**Keywords:** cancer-associated fibroblasts, cancer cell-derived exosomes, invasive cancer cells, cancer cell invasion, 3D microfluidics

## Abstract

Cancer-associated fibroblasts (CAFs) in the cancer microenvironment play an essential role in metastasis. Differentiation of endothelial cells into CAFs is induced by cancer cell-derived exosomes secreted from cancer cells that transfer molecular signals to surrounding cells. Differentiated CAFs facilitate migration of cancer cells to different regions through promoting extracellular matrix (ECM) modifications. However, in vitro models in which endothelial cells exposed to cancer cell-derived exosomes secreted from various cancer cell types differentiate into CAFs or a microenvironmentally controlled model for investigating cancer cell invasion by CAFs have not yet been studied. In this study, we propose a three-dimensional in vitro cancer cell invasion model for real-time monitoring of the process of forming a cancer invasion site through CAFs induced by exosomes isolated from three types of cancer cell lines. The invasiveness of cancer cells with CAFs induced by cancer cell-derived exosomes (eCAFs) was significantly higher than that of CAFs induced by cancer cells (cCAFs) through physiological and genetic manner. In addition, different genetic tendencies of the invasion process were observed in the process of invading cancer cells according to CAFs. Our 3D microfluidic platform helps to identify specific interactions among multiple factors within the cancer microenvironment and provides a model for cancer drug development.

## 1. Introduction

High mortality associated with cancer is attributable to metastasis and uncontrolled spread of cancer cells across the body [1]. Cancer cells detached from the primary site enter the bloodstream and lymphatic systems and are transported throughout the body [2]. Metastatic cells usually colonize the ends of capillaries, undergo extravasation into the surroundings, induce angiogenesis and finally develop as secondary cancer in a foreign environment [3,4].

Most cancer cells utilize the extracellular matrix (ECM), which acts as a physical constraint, to mediate communication signals, promote interactions with other cells and establish the cancer microenvironment [5,6,7,8]. Cancer microenvironments are mainly constituted by cancer-associated fibroblasts (CAFs), macrophages, mesenchymal stem cells and cancer-specific ECM [9].

In particular, CAFs play an important role in cancer cell invasion by mediating the remodeling of ECM leading to secondary metastasis [10,11]. CAFs are the most abundant stromal cell population in the tumor microenvironment, and activated CAFs secrete a high level of growth factors and inflammatory cytokines as a promoter of tumor progression [12]. CAFs can be originated from various cell populations such as activated tissue-resident fibroblasts, trans-differentiated pericytes and adipocytes. CAFs can be also generated from trans-differentiated epithelial and endothelial cells by an epithelial-to-mesenchymal transition (EMT) or an endothelial-to-mesenchymal transition (EndMT) [13]. CAFs are characterized by the loss of cell–cell junctions, and the acquisition of migratory and invasive properties. CAFs induce the upregulation of the enzymes contributing to the degradation of the ECM components, activate integrins that bind ECM receptors and promote the entry of carcinoma cells into ECM [14,15].

EndMT is a transition process from an endothelial phenotype to a mesenchymal phenotype resembling a spindle-like shape, and increases the migration of metastatic cells [16,17]. EndMT contributes to the accumulation of CAFs and most CAFs are formed by EndMT [18]. For example, with regard to the omental tumor microenvironment, omental fibroblasts are trans-differentiated from adipose-derived mesenchymal stem cells by ovarian cancer-derived exosomes and are associated with transforming growth factor beta (TGF-β) signaling secreted by both cancer cells and stromal cells [19,20]. TGF-β contributes to recruiting stromal cell types characterized by CAFs, inducing ECM production and downregulating the expression of ECM-associated protease [21].

Recent studies have reported that cancer cell-derived exosomes considerably induce the endothelial-to-mesenchymal transition (EndMT) and reconstitute pre-metastatic niche, and generate a tumor microenvironment in a distant metastatic site [22,23,24]. Exosomes (vesicles secreted from cells) contain miRNA, mRNA, DNA and signal proteins that act as pivotal communication agents. Exosomes derived from cancer cells deliver messages from the tumor to near or distant tissue sites, and alter the phenotype, functional attributes of target cells to activate angiogenesis, thrombosis, inflammation, immunosuppression and metastasis [25,26]. Exosomes directly trigger EndMT, initiating metastasis through controlling the expression of metastasis-related genes [22,27,28]. Additionally, recent studies have reported that exosomes act as communication signals between primary and secondary cancer invasion sites [29].

However, it is not known whether progress of EndMT by exosomes derived from different cancer cells has a common gene and whether specific physiological phenomena occur through such a common gene. In addition, with the lack of a microenvironmentally controllable engineering tool, it has not been investigated how the genetic factors of CAFs influence the development of secondary cancers and how CAFs interact with invasive cancer cells in real time in a three-dimensional environment.

In this study, we isolated and characterized exosomes from three different cancer cell types: B16BL6 (murine melanoma), A431 (human squamous carcinoma) and MDA-MB-231 (human breast carcinoma). We further investigated the differences in CAFs induced by various cancer cell-derived exosomes and their potential involvement in development of secondary cancer invasion sites within a 3D microfluidic cancer microenvironment in physiological and genetic manner.

Our data from this 3D microfluidic platform showed that CAF-mediated guidance of cancer cell invasion in the extracellular matrix was dependent on physiological factors. Furthermore, data from genetic analyses revealed the common expression of ECM-related genes in all three CAF types. Interestingly, different genetic characteristics were identified in relation to invasiveness of B16BL6, A431 and MDA-MB-231 cell lines. We found that the invasion process is strongly influenced by the interaction of components of the cancer microenvironment, such as cancer cell-derived exosomes, CAFs and ECM. The microfluidic applications in cancer research provide a promising alternative screening platform for anti-cancer drug discovery and delivery and are anticipated to contribute significantly to cancer research programs and accelerate drug development efforts for personalized therapy.

## 2. Results and Discussion

### 2.1. Physiological Interplay between CAFs and Cancer Cells Promotes Cancer Cell Invasion

CAFs contribute to ECM degradation and destruction of the endothelial barrier, and thus they play a role in metastatic growth and progression of cancer cells [30,31]. The differentiation of endothelial cells into CAFs is induced by cancer cells. Recent studies have shown that exosomes derived from cancer cells are able to induce the differentiation of CAFs from endothelial cells [22,24].

To investigate whether exosomes derived from cancer cells are more effective in generating CAFs from HUVECs than cancer cells themselves, (1) HUVECs were cultured in the central channel of the 3D microfluidic device, (2) exosomes derived from cancer cells were injected into the endothelialized central channel for the differentiation of HUVECs into CAFs and (3) after CAFs were generated, cancer cells were injected into the central channel to investigate cancer cell invasion (Figure 6a).

To differentiate HUVECs into CAFs, exosomes were isolated from three different cancer cells (mouse melanoma, human skin cancer cells and human breast cancer cells); since melanoma is a known cause of metastasis, skin cancer is a common invasive cancer with malignant proliferation, and breast cancer is the most common cancer in women. The size distribution of the isolated exosomes determined through NTA analysis was 30–200 nm, which is consistent with the range reported in the literature [32] (Figure S1a–c). The morphological shape of the exosomes observed by using TEM was also consistent with the documented geometric structure [33] (Figure S1d–f).

The isolated exosomes alone or with cancer cells were injected into the device to induce the EndMT process (differentiation of endothelial cells, HUVECs in this study, into CAFs). As shown in Figure 6, HUVECs underwent significant morphological and genetic changes when differentiating into CAFs induced by exosomes or cancer cells: Active filopodia for sprouting and movement into a 3D collagen gel matrix, as well as the expression of fibroblast-specific protein (FSP-1), a representative CAF marker.

We found that in the absence of CAFs cancer cells rarely move towards the collagen matrix (Figure 6a,b). Only when HUVECs were differentiated into CAFs by either exosomes (eCAFs, Figure 6e,f), cancer cells (cCAFs, Figure 6c,d) or both exosomes and cancer cells (ecCAFs, Figure 6g,h), cancer cells could migrate into the collagen matrix regardless of cancer cell type. Interestingly, ecCAFs were able to induce the aggressive penetration of cancer cells into the 3D collagen gel (Figure 6g,h), and were more effective than eCAFs (Figure 6e,f) and cCAFs (Figure 6c,d). Recent studies have reported that exosomes extracted from human gastric cancer cells promoted cell growth by ~2 fold and induced pericyte-to-CAFs transition. This showed that exosomes could induce the transition at high efficiency [34]. These results represent that CAFs differentiated from HUVECs by exosomes or cancer cells are essential in the metastatic migration of cancer cells.

**Figure 1:**
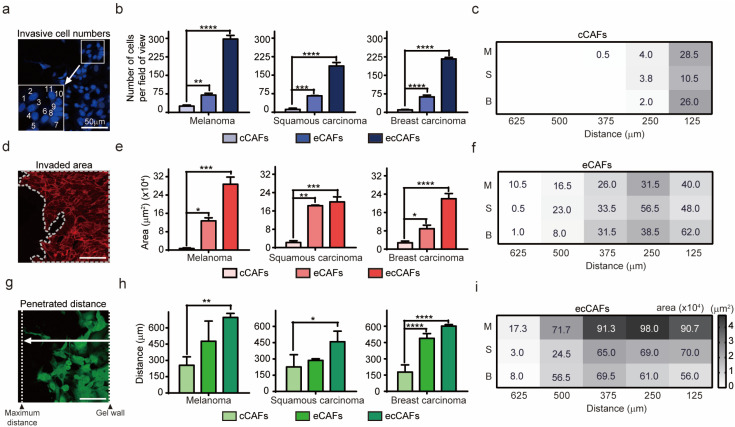
Physiological evidence of CAF-induced invasion. (**a**,**d**,**g**) Schematic diagram showing the measurement analysis process of cancer cell invasion. Scale bar: 50 μm. (**b**,**e**,**h**) Comparison of the number, distance, area and invasiveness of the three different cancer cell types. (**c**,**f**,**i**) Analysis of invasiveness based on area of invasive cells in relation to distance travelled into the ECM matrix. Larger areas are represented by darker color. The numbers indicate the degree of invasiveness of cells (M: Melanoma; S: Squamous carcinoma; B: Breast carcinoma). Data are presented as means ± SEM (* *p*-value < 0.05, ** *p*-value < 0.01, *** *p*-value < 0.001, **** *p*-value < 0.0001).

To quantitatively analyze the invasiveness of cancer cells, we counted the number of cancer cells in the collagen gel (invasive cell numbers), measured the area of propagated cells (invaded area) and measured the maximum distance travelled from the primary site (penetrated distance). As shown in Figure 1, ecCAFs always showed a higher number of invasive cells (Figure 1a–c), larger invaded areas (Figure 1d–f) and longer penetrated distances (Figure 1g–i) than eCAFs and cCAFs. Moreover, eCAFs always showed more effective migration of cancer cells than cCAFs.

This phenomenon may be explained as the role of CAFs induced by exosome-mediated stimulation in the invasive and invasion potencies of cancer cells [35]. Each invasive cancer cell type had unique invasion characteristics in microfluidic model cultured with ecCAFs, even if there were no differences in the extent of invasion when differentiating cCAF (Figure 1c,f,i). eCAF with B16BL6 cells travelled the farthest distance from the primary site. B16BL6 cancer cells not only invaded the farthest but were also produced at the highest proliferation rate (Figure 1i). While the number of A431 cells invaded by eCAF was less than that of B16BL6 cells, A431 cells generated a higher number of CAFs and a larger area of differentiation compared to the distance penetrated (Figure 1f,i).

Melanoma cells are highly metastatic and capable of penetrating the reconstituted basement membrane [36,37]. Malignant melanoma cells possess a significantly higher mortality than nonmelanoma skin cancer cells, but the extent of spread of invasive squamous cells, such as A431, depends on the cancer size and degree of differentiation [38,39,40]. MDA-MB-231 (human breast cancer cells) ecCAFs produced cells that travelled a comparatively long distance relative to number of cells and area occupied by cells (Figure 1c). Metastatic breast cancer has recently been shown to develop at a distance from the primary site in organs, such as brain, liver, lung or bones [41,42]. In our experiments, invasiveness of differentiated CAFs induced by cancer cell-derived exosomes was significantly higher than that of CAFs induced by cancer cells (Figure 1). Cancer cell-CAF crosstalk has been shown to promote growth and invasion of specific cancer cell types. CAFs have the ability to breach the matrix and promote invasiveness of cancer cells [43,44]. Our results support greater progression of ecCAF invasion relative to that of cCAFs, consistent with in vivo findings, validating the critical role of CAFs in cancer invasion.

### 2.2. Genetic Analysis of HUVEC Differentiation into CAF by Exosomes

To identify mRNAs differentially expressed in eCAFs (differentiated HUVECs by exosomes derived from cancer cells), we used the Nanostring nCounter platform for profiling mRNA expression and the platform can measure the expression levels of 770 genes [45,46]. The genes are involved in the cancer progression process such as angiogenesis, extracellular matrix remodeling (ECM), epithelial-to-mesenchymal transition (EMT) and metastasis. Exosomes were extracted from the three different cancer cells (B16BL6, A431 and MDA-MB-231). The eCAFs were acquired from the microfluidic device after incubating HUVECs and the exosomes for three days. The mRNA profiles of three different eCAFs generated by the respective exosomes were analyzed. As a control, the expression levels of the 770 mRNAs in HUVECs were also measured. It should be noted that it is impossible to separate CAFs from HUVECs and therefore the mRNA levels were measured using the mixture of eCAFs and HUVECs assuming that DEGs in CAFs can be identified by using non-differentiated HUVECs as a control.

**Figure 2:**
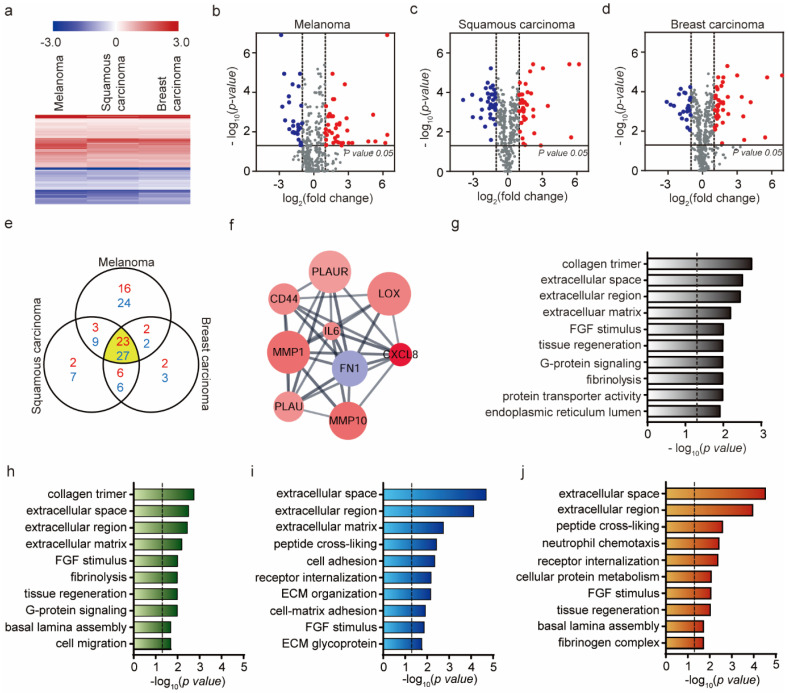
Identification of differentially expressed genes from eCAFs and their biological functions. (**a**) Hierarchical clustering of expression profiles of differentially expressed mRNAs among three eCAFs (the eCAFs were produced by the exosomes extracted from B16BL6, A431 and MDA-MB-231 cells) (*p*-value < 0.05). Red color indicates high relative expression and blue indicates low relative expression. (**b**–**d**) Volcano plot showing gene expression differences among the three eCAFs. Red, DE genes with log_2_ (fold change) > 1; blue, DE genes with log_2_ (fold change) < −1. (**e**) Venn diagram showing differentially expressed overlapping gene numbers for three eCAFs. The number of overlapping regions shows the largest number of differentially expressed genes. Red represents log_2_ (fold change) > 1 and blue represents log_2_ (fold change) < −1. (**f**) Top module of the protein–protein interaction (PPI) network for densely connected nodes. Red, DE genes with log_2_ (fold change) > 1; blue, DE genes with log_2_ (fold change) < −1. Larger node size is associated with a more significant *p*-value. (**g**) Gene ontology (GO) term enrichment analysis of common mRNA expression in three eCAFs (*p*-value < 0.05, **|** log_2_ (fold change) **|** > 1). (**h**–**j**) Gene ontology (GO) term enrichment analysis of expressed mRNA; B16BL6, A431, MDA-MB-231 cells (*p*-value < 0.05, **|** log_2_ (fold change) **|** > 1). The dashed line signifies *p*-value of 0.05.

As shown in Figure 2a, the three eCAFs show very similar gene expression profiles in the 770 genes. Though the triggering exosomes were derived from three different cancer cells, the exosomes would contain the same or very similar genetic components (mRNA, proteins, etc.) to induce the EndMT (the differentiation of HUVECs into CAFs in this study). Consistently, most of the identified DEGs overlap regardless of cancer cell types (23 up-regulated genes and 27 down-regulated genes) (Figure 2e). The Venn diagram shows the numbers of overlapping and non-overlapping differentially expressed genes (DEGs) in the three different eCAFs. For this identification, the DEGs showing a high change in expression levels (|log_2_ (fold change)|>1) and statistical significance when compared with the levels in HUVECs (*p*-value < 0.05) were selected, and the selected DEGs are shown in volcano plots in respect to the cancer cell type (Figure 2b–d and Figure S2a).

The overlapping 50 genes (23 up-regulated and 27 down-regulated DEGs) in Figure 2e were further analyzed. Firstly, the protein–protein interaction (PPI) network of the 50 DEGs was constructed using STRING PPI database [27924014] and the network was analyzed by using Cytoscape and MCODE [12525261] to identify core clusters. As shown in Figure 2f, a highly inter-connected cluster was identified. Of these nodes, IL-6 exhibited the highest degree of connectivity. Consistent with our result, IL-6 secreted from CAFs are known to increase the signaling for EMT and EndMT [47,48]. The other genes in the cluster are also capable of further driving the differentiation of HUVECs into CAFs and extracellular matrix degradation [49]. Specifically, these genes induce growth and pro-inflammatory factors, in turn promoting the formation of a pro-metastatic microenvironment. Activated CAFs secrete excessive metalloproteinases (MMP), lysyl oxidase (LOX) and plasminogen activator (PA), leading to degradation of ECM, construction of basement membrane, ECM remodeling and generation of a stiff environment that facilitates the metastatic process (Figure S2b–d) [50,51,52].

To get into broad insight on the overlapping 50 DEGs, we also performed GO enrichment and KEGG pathway enrichment analyses. As shown in Figure 2g, the enriched GO terms are associated with ECM such as collagen trimmer (*p*-value = 0.0018), extracellular space (*p*-value = 0.0031), extracellular region (*p*-value = 0.0036) and extracellular matrix (*p*-value = 0.0065). Notably, ECM-associated proteins and their receptors are related to degradation and facilitation of cell movement across the matrix or processing or deposition of the matrix [49]. For these processes to occur, MMPs and PA are secreted, which promote cell penetration through the basement membranes, eventually initiating invasion [53,54]. GO enrichment analyses were further performed with the DEFs of the three respective eCAFs (Figure 2h–j). ECM-related GO terms are common in the DEGs of the three eCAFs. However, there are small differences in the enriched GO terms. For example, both FGF-mediated signaling and G-protein signaling were significantly enriched within the DEGs of eCAFs triggered by mouse melanoma exosomes (Figure 2h). However, only FGF-mediated signaling was significantly enriched within the DEGs of eCAFs triggered by human cancer cell exosomes (skin and breast cancer cells, Figure 2i,j, respectively). This may represent that ECM-related function and FGF-mediated signaling are essential in the eCAF function, but there could be slight difference in physiological features depending on the origin of exosomes.

The number of eCAFs was recorded over time and compared between the three types of cancer cell-derived exosomes (Figure S3a–d). The number of eCAFs increased up to three days, followed by a decrease, but the number of eCAFs was not significantly different among cancer cell lines. There were no statistically significant differences found either in terms of physiological phenomenon such as the rate of CAFs differentiation or in terms of genetic characteristics. However, these genetic analyses identified common expression of an ECM-related gene in all three types of CAFs, which might have been responsible for the similar physiological phenomenon observed in all three CAFs.

### 2.3. Genetic Analysis of HUVEC Differentiation into CAF by Cancer Cells

To investigate the genetic alterations in cCAFs (differentiated HUVECs triggered by cancer cells), we also measured the gene expression levels of cCAFs induced by three respective cancer cells (mouse melanoma, human skin cancer cells and human breast cancer cells) and the expression levels were compared with those in HUVECs. It should be noted that it is impossible to isolate cCAFs from the mixture of HUVECs, cCAFs and cancer cells. Therefore, the DEGs obtained from the mixture of the three cell types should be carefully interpreted.

**Figure 3:**
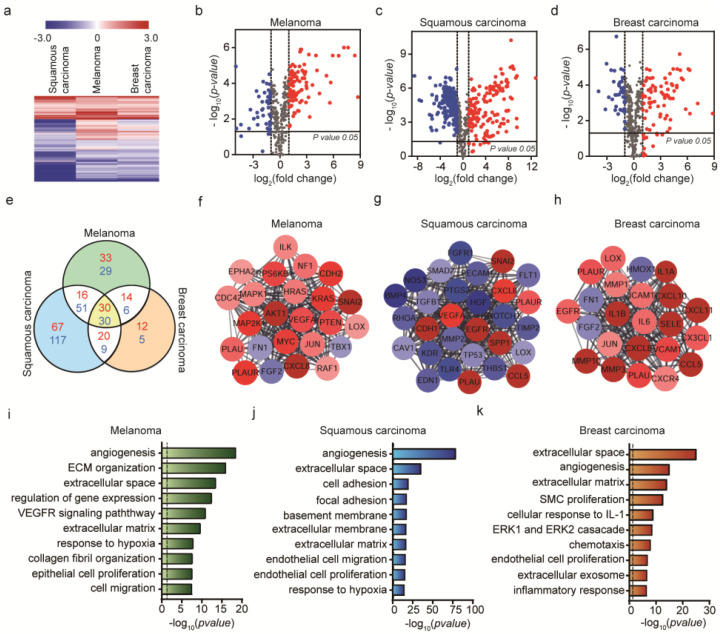
Identification of differentially expressed genes from cCAFs and their biological functions. (**a**) Hierarchical clustering of expression profiles of differentially expressed mRNAs among the three cell lines (*p* < 0.05). Red color indicates high relative expression and blue indicates low relative expression. (**b**–**d**) Volcano plot showing gene expression differences among the three cell lines, with red representing DE genes with log_2_ (fold change) > 1 and blue representing DE genes with log_2_ (fold change) < −1. (**e**) Venn diagram showing the significant gene numbers for the three cancer cell lines. Red represents log_2_ (fold change) > 1 and blue log_2_ (fold change) < −1. Comparison of DE gene expression levels with cCAFs and HUVECs. (**f**–**h**) Top module of the protein–protein interaction (PPI) network for densely connected nodes. Red, DE genes with log_2_ (fold change) > 1; blue, DE genes with log_2_ (fold change) < −1. Larger node size represents more significant *p*-values. (**i**–**k**) Gene ontology (GO) term enrichment analysis of mRNA expression in B16BL6, A431 and MDA-MB-231 cells (*p*-value < 0.05, **|** log_2_ (fold change) **|** > 1). The dashed line signifies a *p*-value of 0.05.

As shown in Figure 3a, the expression profiles of the 770 genes are very different depending on the type of cancer cells used. In eCAFs, the expression profiles of eCAFs induced by three different exosomes were very similar regardless of the exosome origin of cancer cells. This could be because the exosomes excreted from cancer cells contain a small number of genetic materials that are required to differentiate HUVECs into CAFs, while cancer cells secrete other proteins to control the microenvironment and some of them induce the differentiation of HUVECs to CAFs. In short, cCAFs were differentiated by many factors excreted from cancer cells including exosomes, and as a result the expression profiles of cCAFs could become different depending on the cancer cells used. Consistently, the number of DEGs common in the three different cCAFs is only nine (eight up-regulated and one down-regulated genes) (Figure 3e). The volcano plots of the DEGs in the three cCAFs are shown in Figure 3b–d. In the figure, DEGs that showed |log_2_(fold change)|>1 and *p*-value < 0.05 when compared with the expression levels obtained from HUVECs (control) are colored in red (up-regulated) and blue (down-regulated).

To investigate which cellular processes in cCAFs were affected by the respective cancer cells, we constructed respective PPI networks of the DEGs identified. The core clusters identified from the three different cCAFs are shown in Figure 3f–h. For mouse melanoma (Figure 3f), the cluster is composed of only up-regulated genes, and most of them are involved in signal transduction, specifically AKT1 pathway. This represents that mouse melanoma activates the signal transduction function in cCAFs. For human skin cancer cells, the cluster was related to VEGFA, EGFR, etc. CAFs were shown to promote angiogenesis by regulating the tumor microenvironment by the expression of VEGF and EGF [55]. For breast cancer cells, the cluster is composed of many interleukin proteins such as IL-1, IL-6, etc. CAFs provide cancer invasion by secreting cytokines, and these cytokines have been suggested as critical tumor microenvironment factors [56].

The enriched GO terms within the DEGs of three cCAFs were analyzed (Figure 3i–k). In all cCAFs, ECM-related functions were significantly enriched. Interestingly, in all cCAFs angiogenesis was significantly affected by the cancer cells, which was not identified enriched in eCAFs. Exosomes and cancer cells are able to induce ECM-related functions in CAFs, but cancer cells may have exclusive factors that induce the formation of blood vessels (angiogenesis).

### 2.4. Genetic Analysis of HUVEC Differentiation into CAF by Both Exosomes and Cancer Cells

Lastly, we investigated the effect of both exosomes and cancer cells on the differentiation of HUVECs into CAFs. The exosomes extracted from the three different cancer cells were injected into the microfluidic device and the cancer cells were also injected into the device. The difference from eCAF experiments was that cancer cells were additionally supplied. After incubation of HUVECs with exosomes and cancer cells within the device, the cell mixture was extracted and the expression levels of the 770 genes were analyzed. It should be also noted that the mRNAs were extracted from the mixture of ecCAFs, undifferentiated HUVECs and cancer cells, and thus the expression profiles should be carefully interpreted. In this analysis, the expression levels of mRNAs in eCAFs were used as a control.

**Figure 4:**
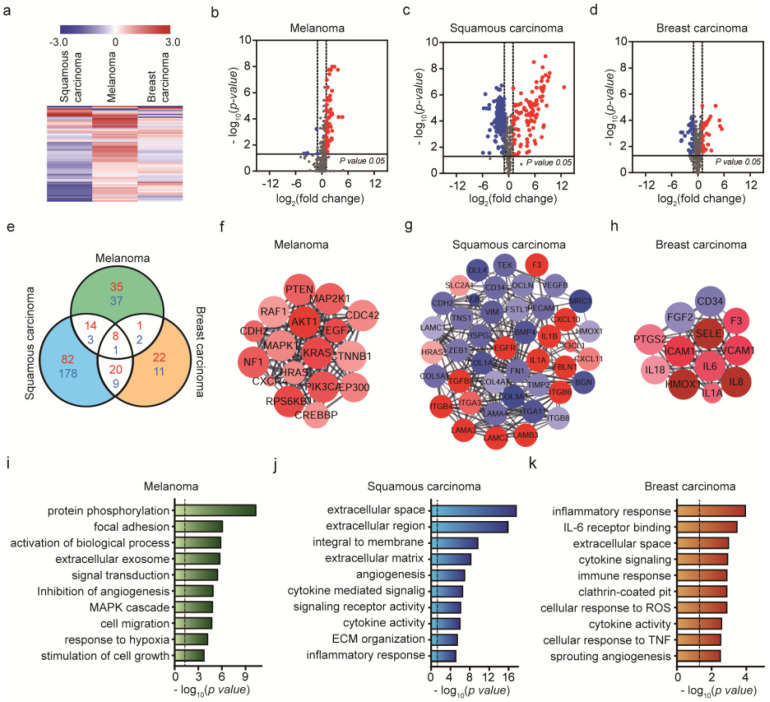
Identification of genes and functions associated with cancer invasion model created by the exosome-induced CAFs (ecCAFs). (**a**) Hierarchical clustering of expression profiles of invasiveness of cancer cells created by the exosome-induced CAFs (*p*-value < 0.05). Red color scale indicates high relative expression and blue indicates low relative expression. (**b**–**d**) Volcano plot showing gene expression differences among the three cell lines, with red representing DE genes with log_2_ (fold change) > 1 and blue representing DE genes with log_2_ (fold change) < −1. (**e**) Venn diagram showing the significant gene numbers for the invasiveness of cancer cells. Comparison of DE gene expression levels with ecCAFs and eCAFs. Red represents log_2_ (fold change) > 1 and blue log_2_ (fold change) < −1. (**f**–**h**) Top module of protein–protein interaction (PPI) network for densely connected nodes. Red, DEs with log_2_ (fold change) > 1; blue, DEs with log_2_ (fold change) < −1. Larger node size represents more significant *p*-values. (**i**–**k**) Gene ontology (GO) term enrichment analysis of mRNA expression in B16BL6, A431 and MDA-MB-231 cells (*p*-value < 0.05, **|**log_2_ (fold change)**|** > 1). The dashed line signifies a *p*-value of 0.05.

Clusters of expression profiles were different among the cell groups (Figure 4a). Venn diagrams of DE genes for each cancer cell line were generated (Figure 4e). Among a total of 770 genes, we identified 23% upregulated and 35% downregulated genes in A431 cells, compared to <10% upregulated and downregulated genes in B16BL6 and MDA-MB-231 cells during cancer invasion based on the Volcano plot (Figure 4b–d). Using the protein–protein interaction (PPI) network, where the nodes and edges were constructed based on significant expression levels (*p*-value < 0.05, **|** log_2_ (fold change) | > 1), key genes and important gene modules were identified. The mouse melanoma induced the up-regulation of signal transduction-related genes including ‘signal transduction (*p*-value = 0.00000001)’ and ‘MAPK cascade (*p*-value = 0.00037)’. Human skin cancer cells altered the functions related to ‘angiogenesis (p-value = 0.000000046)’, ‘cytokine-mediated signaling (*p*-value = 0.00000028)’, etc. Human breast cancer cells also altered the genes related to ‘cytokine signaling (*p*-value = 0.00012)’, ‘immune response (*p*-value = 0.0001), etc. AKT1 was identified as the gene with the highest degree of connectivity in B16BL6 cells, EGFR in A431 cells and IL-6 in MDA-MB-231 cells (Figure 4f–h). As shown in Figure 4f, the selected genes of B16BL6 cells were involved in promoting melanoma development in MCODE analysis and the enriched genes identified through GO enrichment involved in MAPK cascade and PI3K-Akt signaling (Figure 4i). Cutaneous melanoma commonly arises from transformation of melanocytes through accumulation of mutations in the BRAF gene, which activate the MAPK/Extracellular signal-regulated Kinase (ERK) signaling pathway [57]. BRAF-mutant melanoma cells respond to BRAF inhibitor by PLX4720, but rapid reactivation of ERK/MAPK. CAFs have been shown to tolerate BRAF inhibition in areas of high stroma. The PLX4720 has an effect on the tumor stroma and promote matrix production, ECM remodeling leading to activation of integrin β1/focal adhesion kinase (FAK)/Src and ERK signaling. Melanoma-associated fibroblasts activate paradoxically adhesion-mediated signaling, which supports residual disease, result in enhancing the population of cancer cells [20,58,59]. Activating mutations in kinase PI3K/AKT have been reported that increase AKT activity, promoting melanoma and resistance to apoptosis [60]. In addition, overexpression or mutational activation of receptor tyrosine kinase (RTK) is known to cause hyperactivation of RAS leading to upregulated MAPK or PI3K/AKT signaling in resistant melanoma cells [61]. High levels of MAPK and PI3K typical in melanoma cells enhance anti-apoptotic genes while suppressing the expression of pro-apoptotic genes [57].

The genes for squamous carcinoma were associated with migration-related integrin in MCODE analysis (Figure 4g) and the relationship between integrin and extracellular matrix confirmed through GO enrichment analysis (Figure 4j). In human squamous carcinoma cells (SCC) of the skin, epidermal growth factor receptor (EGFR) and α6β4 integrin overexpression are associated with migration, invasion and disruption of hemidesmosomes in the extracellular matrix (ECM) through crosstalk with Src family kinase (SFK) (Figure 4g) [62]. Integrins are the major receptors required for cell adhesion in response to extracellular cues and co-operate with cell surface receptors and growth factors or cytokines to regulate intracellular pathways. Furthermore, α6β4 integrin bound to laminin-332 ligand in the matrix disrupts cell–cell adhesions and triggers scattering of SCC. In particular, interactions of laminin-332 (containing α3, β3 and γ2 chains) and α6β4 integrin bound to collagen VII modulate EGFR activation, in turn, driving SCC carcinogenesis, malignant cancer progression and invasion.

In breast cancer, IL-6 receptor involved in the inflammatory response was enriched in GO analysis of ecCAFs induced by MDA-MB-231 cells, as shown in Figure 4k. IL-6 is a major proinflammatory cytokine involved in multiple processes including cell cycle progression, suppression of apoptosis and survival of DNA-damaged cells. Cytokines involved in cancer-related inflammation promote malignant progression [63,64]. IL-6 expresses various adhesion molecules, such as ICAM-1, VCAM-1 and E-selectin, which play major roles in tethering of cancer cells to the endothelial wall [65,66]. IL-6 has been reported to induce cancer cell extravasation and destruction of the basement layer to facilitate progression of metastasis [65].

Additionally, GO enrichment analysis thus provides comprehensive information on the different characteristics of invading cancer cells, and detailed insights into mRNA-mediated mechanisms. Determination of protein interactions presented an efficient approach for screening hub genes of B16BL6, A431 and MDA-MB-231 cells (Figure 4f–h).

To investigate the effect of exosomes on the differentiation of HUVECs into CAFs, we compared the expression levels of mRNAs in ecCAFs vs. cCAFs. In this case, the only difference is the addition of exosomes and thus it is appropriate to investigate the effect of exosomes on CAFs.

**Figure 5:**
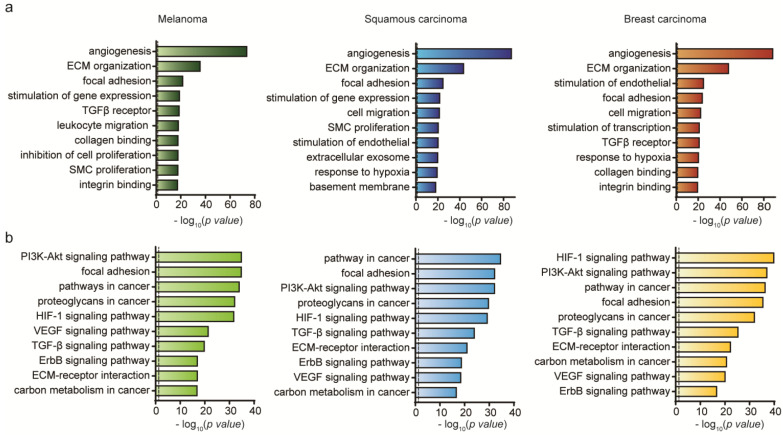
Comparative expression profiling of cancer invasion between ecCAFs and cCAFs. (**a**) Gene ontology (GO) term enrichment analysis of differentially expressed mRNAs in B16BL6, A431 and MDA-MB-231 cells (*p*-value < 0.05, | log_2_ (fold change) | > 1). (**b**) KEGG pathway analysis of differentially expressed mRNAs in B16BL6, A431 and MDA-MB-2321 cells (*p*-value < 0.05, |log_2_ (fold change)| > 1). The dashed line signifies a *p*-value of 0.05.

To get broad insight into the effect of exosomes on CAFs, we performed enrichment analysis of GO terms (Figure 5a) and KEGG pathways (Figure 5b) within the DEGs in ecCAFs compared with cCAFs. In GO term analysis, the terms (angiogenesis, ECM organization and focal adhesion) were commonly enriched within ecCAFs. We also found that other terms related to TGF-beta signaling, SMC proliferation, response to hypoxia, etc. were also enriched within two of the ecCAFs. This result represents that exosomes are more capable of activating cellular functions required for cancer cell migration. KEGG pathway enrichment analysis also shows consistent results with GO enrichment analysis. For example, the pathways such as focal adhesion, ECM-receptor interaction and TGF-beta signaling pathway were also enriched within the DEGs (Figure 5b).

CAFs can be originated from various cell populations; in particular, epithelial cells and endothelial cells were important source of CAFs through the EMT and EndMT. Our findings indicate that EndMT-triggered ecCAFs in the 3D system used in this study reproduce in vivo invasive progression. The variable properties of cCAF differentiation were attributable to cancer cells, because the DE gene levels of cCAFs from B16BL6, A431 and MDA-MB-231 cell lines show a similar trend to the gene levels of cCAFs rather than eCAFs, and were verified from a genetic perspective. The DE gene levels of ecCAFs showed enhanced angiogenesis and focal adhesion-related signaling pathways compared to cCAFs. Further research is needed to investigate clinical studies of CAFs and cancer invasion for critical signaling pathway and anti-metastasis therapies.

## 3. Materials and Methods

### 3.1. Cultivation of Cancer Cells

B16BL6 murine melanoma cells (KCLB No. 8006; Korean Cell Line Bank [KCLB], Seoul, Korea) were cultured in minimum essential media alpha (α-MEM; Gibco, Waltham, MA, USA) supplemented with 10% (*v/v*) fetal bovine serum (FBS; Rocky Mountain Biologicals, Missoula, MT, USA), 1% (*v/v*) penicillin and streptomycin (Lonza, Basel, Switzerland) on culture dishes. A431 human squamous carcinoma cells (CRL-1555; American Type Culture Collection (ATCC), MD, Manassas, VA, USA) and MDA-MB-231 human breast carcinoma cells (HTB-26; ATCC, MD, Manassas, VA, USA) were cultured in Dulbecco’s modified eagle medium (DMEM; Lonza) supplemented with 10% (*v/v*) fetal bovine serum (Rocky Mountain Biologicals, Missoula, MT, USA), 1% (*v/v*) penicillin and streptomycin (Lonza) on culture dishes. Primary human umbilical vein endothelial cells (HUVECs; CC-2517; Lonza) were cultured in endothelial cell growth medium (EGM-2; Lonza) supplemented with 10% (*v/v*) fetal bovine serum (Rocky Mountain Biologicals, Missoula, MT, USA) and 1% (*v/v*) penicillin and streptomycin (Lonza) on culture dishes. All cells were incubated at 37 °C under 5% CO_2_.

### 3.2. Isolation of Cancer Cell-Derived Exosomes

Cancer cells were cultured in the respective media until 80% confluence. Medium containing 10% FBS was replaced with an FBS-depleted medium (dFBS; System Biosciences, Mountain View, CA, USA) and cells were allowed to grow for 24 h. This step was performed to specifically obtain exosomes secreted by cancer cells and not those of FBS-containing medium. Cells were cultured for 24 h with dFBS and centrifuged at 500 *g* for 10 min to remove cell debris. The collected supernatant was transferred to a new flask and re-centrifuged at 5000 *g* for 30 min. After final collection, the supernatant was centrifuged at 10,000 *g* for 30 min. Subsequently, 30 mL supernatant was added to 6 mL solution of the ExoQuick-TC kit (System Biosciences, Palo Alto, CA, USA) within a new conical flask and proper mixing of the contents was ensured. The conical tube was refrigerated at 4 °C in an upright position for over 12 h, followed by centrifugation of the mixture at 1500 *g* for 30 min. The supernatant was aspirated and the remaining mixture was collected for centrifugation at 1500 *g* for 5 min. Following complete aspiration of the supernatant, the pellet was re-suspended in 500 μL phosphate-buffered saline (PBS; Lonza). The suspension was collected using a 1 mL syringe and filtered through a 0.2 μm syringe filter with a diameter of 4 mm (Corning, Corning, NY, USA) to obtain exosomes. All centrifugation and refrigeration steps were conducted at 4 °C.

### 3.3. Characterizations of Exosomes

Exosome samples were imaged under a JEM-1400 Plus transmission electron microscope (JEOL Ltd., Tokyo, Japan) at an under focus of 0.8–1.5 μm and recorded using an UltraScan OneView CMOS camera (Gatan, Pleasanton, CA, USA). Samples were prepared by loading 5 μL solution onto an EM grid covered with glow-discharged continuous carbon film. The grid was washed with deionized water after 1 min and stained with 1% uranyl acetate for 1 min. After removal of staining solution using filter paper, the grid was dried completely in open air.

The size distribution of particles was determined by nanoparticle tracking analysis (NTA), which assesses the combined properties of light scattering and Brownian motion. Isolated EVs in liquid were diluted in 1 mL phosphate-buffered saline (PBS; Lonza), and visualized and counted by a Nanosight instrument (Malvern Instrument, Worcestershire, UK) at a temperature of 25 °C using a 488 nm laser.

### 3.4. Preparation of 3D Microfluidic Cancer Microenvironment

The 3D microfluidic TME was created by injecting collagen into the required channels of the microfluidic device. The collagen gel solution was prepared by mixing four components in the following order: Collagen (8.9 mg/mL, rat tail collagen type I, high concentration; BD Biosciences, Palo Alto, CA, USA), 10× PBS with phenol red (Thermo Scientific, Waltham, MA, USA), 0.5 N NaOH and distilled deionized water. The concentration of the working collagen gel solution was 5 mg/mL, and pH was adjusted to 7.4 using 0.5 N NaOH. The gel-filling region of the microfluidic device was slowly filled with collagen and left to harden at 37 °C for 30 min. Subsequently, all ports were filled to the brim with endothelial cell growth medium-2 (EGM-2; Lonza) [22].

### 3.5. Culturing of HUVECs in Microfluidic Devices

**Figure 6:**
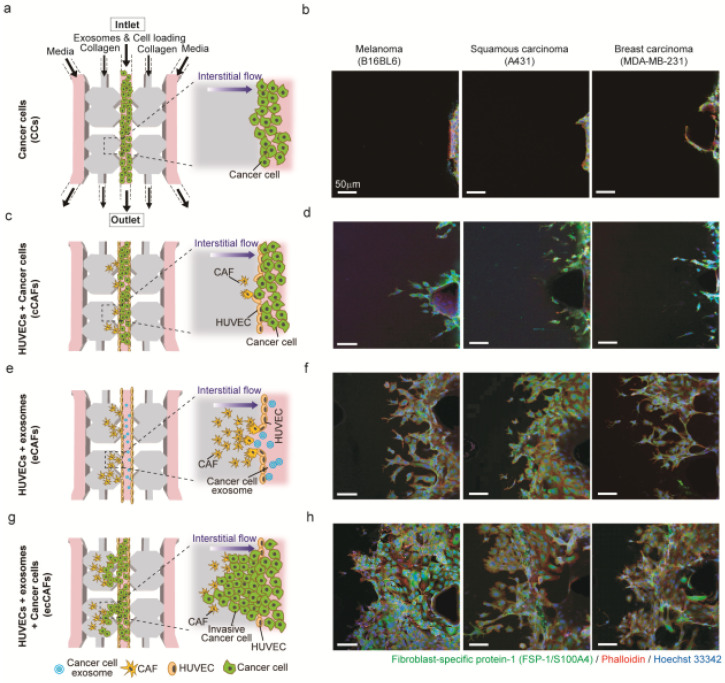
Three-dimensional microfluidic model for cancer cell invasion. (**a**,**c**,**e**,**g**) Schematic diagram showing the progression of cancer invasion; (**b**,**d**,**f**,**h**) confocal images of only cancer cells (CCs) free of human umbilical vein endothelial cells (HUVECs), differentiated cancer-associated fibroblasts (CAFs) induced by cancer cells (cCAFs) or cancer cell-derived exosomes (eCAFs), and cancer invasion created by the exosome-induced CAFs (ecCAFs) from melanoma (B16BL6), squamous carcinoma (A431) and breast carcinoma (MDA-MB-231) cells. This 3D microfluidic model includes an endothelial monolayer composed of HUVECs, CAFs (yellow) induced by cancer cell-derived exosomes (blue) and 3D collagen matrix (gray). Then cancer cells (green) were injected to develop a cancer invasion model. Scale bar: 50 μm.

Our microfluidic device was fabricated as previously described [22]. The device consisted of five injection ports (Figure 6a): Two ports fill the channels with collagen gel, two ports are connected to the side channels to induce interstitial flow and one port is connected to the central channel to inject HUVECs or cancer cell-derived exosomes.

HUVECs (Lonza CC-2517) specifically obtained from passages 3 to 4 were cultured in the microfluidic device. After collagen injection, HUVECs (50 µL of 5 × 10^6^ cells/mL) were injected into the central channel in our microfluidic device. All inlet ports including the media and cell loading channels in Figure 6a were filled with the EGM-s medium to provide the nutrients to the HUVECs in the central channel in the microfluidic device. HUVECs were incubated for two days to allow the formation of an endothelial monolayer in the central channel [22].

### 3.6. Cultivation of Cancer-Associated Fibroblasts (CAFs) in the Microfluidic Device

To compare EndMT-triggered CAF generation by applying exosomes or cancer cells themselves as two different stimuli to HUVECs, we cultured CAFs in two methods. (1) cCAFs: After culture of HUVECs for two days within the microfluidic device for the formation of the endothelial monolayer inside the central microchannel, 20 μL of 5 × 10^5^ cells/mL cancer cells were injected into the central channel. (2) eCAF: After culture of HUVECs for two days within the microfluidic device, 10 μL of cancer cell-derived exosomes in 50 μg/mL were injected into the central channel. Only ports on one side of the device were filled to the brim with medium. This difference in the height of the medium between the two sides of the device caused flow in one direction down the pressure gradient, mimicking in vivo interstitial flow between endothelial cells. The EGM-2 medium was replaced every 12 h to maintain the height difference between the ports.

### 3.7. Cultivation of Invasive Cancer Cells in Microfluidic Device

To develop a cancer cell invasion model in a microfluidic device, we injected cancer cell-derived exosomes to induce differentiation of endothelial cells into CAFs, which move towards the collagen matrix adjacent to an endothelialized microchannel. After incubation of CAFs for two days, the cancer cells of the same origin (20 μL of 5 × 10^5^ cells/mL) were injected into the central channel. To generate the height difference of media, medium ports on one side of the device were filled with medium. The EGM-2 medium was replaced every 12 h to maintain the height difference between the ports.

### 3.8. Immunostaining of Cells

Cultured cells in the microfluidic device were mixed with 4% (*w/v*) paraformaldehyde in all reservoirs for fixation and permeabilized with 0.15% (*w/v*) Triton-X 100 for 30 min. Reservoirs were blocked with 3% (*w/v*) bovine serum albumin (BSA; A2153, Sigma, St. Louis, MI, USA) for 1 h, followed by overnight incubation with fibroblast-specific protein-1 antibody (FSP-1; 1:200, Thermo Scientific, Waltham, MA, USA). Subsequently, Alex 488-conjugated goat anti-rabbit IgG secondary antibody (1:400, Thermo Scientific, Waltham, MA, USA) was added and incubated for 3 h, followed by phalloidin-tetramethylrhodamine B isothiocyanate (1:200, Sigma Aldrich, St. Louis, MO, USA) for 2 h. Cell nuclei were stained with Hoechst 33342 (1:500, Thermo Scientific, Waltham, MA, USA) and observed under a confocal laser scanning microscope (LSM700; Carl Zeiss, Jena, Germany)

### 3.9. Quantification of Invasive Cell Number, Invaded Area and Penetrated Distance of CAFs and Cancer Cells

We used the confocal micrographs of Hoechst 33342-stained nuclei and Phalloidin-stained actin filaments to count the number of nuclei and to measure the invaded area by using the ImageJ software. The images were converted to 8-bit images, and the cells in the image were analyzed by the default methods of “Make binary” and “Analyze particle”. Then, the values from the “Count” and the “Total area” displayed in the result table were used. To measure the maximum migration distance of CAFs and cancer cells, we used confocal micrographs of S100A4-stained cytoplasm. We drew a straight line from the gel wall to the end of filopodia of CAFs as a maximum distance by using ImageJ. A *p*-value < 0.05 was considered statistically significant.

### 3.10. RNA Extraction and NanoString nCounter Assay

Total RNA was isolated from cells using MasterPure Complete DNA/RNA Purification Kit (Lucigen-Epicentre^TM^) following the manufacturer’s protocol. The concentration of extracted RNA was determined using a DS 11 Spectrophotometer (Denovix Inc., Wilmington, DE, USA). The total RNA concentration for samples > 100 ng was used. In addition, RNA quality was measured using Fragment Analyzer (Advanced Analytical Technologies, Oak Tree Ct, Ankeny, IA, USA). For analyzing mRNA expression, the NanoString nCounter assay (Nanostring Technologies, Seattle, WA, USA) was used with Pancancer Progression Panel Kit according to the manufacturer’s protocol. All tags were removed, except DNA tags ligated onto the 3′-end of each miRNA in samples. Following identification of unique miRNAs, samples were hybridized with tag-specific nCounter capture and barcoded reporter probes. Hybridization reactions were performed with 5 mL of 5× diluted sample preparation reaction solution. Total RNA samples (100 ng) were incubated at 64 °C for a minimum of 18 h. All excess capture and reported probes were subsequently removed. Each fluorescent barcode was counted using the nCounter digital analyzer to quantify and classify target RNA molecules within the sample.

### 3.11. Module Analysis of the Protein–Protein Interaction (PPI) Network

Cytoscape was applied to analyze gene interactions, expressed as fold change of differential expression (DE), in color and significant genes according to node size. Protein–protein interaction networks were analyzed using molecular complex detection, which is a novel graphic theoretic clustering algorithm that detects densely connected regions within protein networks. Degree cutoff of 2, node score cutoff of 0.2, k-core of 2 and max depth of 100 were set as the parameters in MCODE.

### 3.12. GeneOntology (GO) and Kyoto Encyclopedia of Genes and Genomes (KEGG) Pathway Analyses

Enrichment analysis consisting of a biological process, cellular component and molecular function was performed using the Gene Ontology (GO) analysis tool. The KEGG pathway enrichment was additionally conducted to determine the significant pathways of differentially expressed genes (with *p*-values < 0.05 and gene fold changes > 2) using the KEGG database as the reference.

## 4. Conclusions

In this study, we developed a 3D microfluidic model for cancer invasion with differentiated CAFs to detect cancer cell invasion in real time. The invasiveness of cancer cells in the presence of CAFs induced by exosomes (ecCAFs) was higher than that of CAFs induced by cancer cells (cCAFs) themselves. Our data suggest that CAFs guide invading cancer cells towards the direction of the extracellular matrix and play an important role in cancer cell invasion. In our in vitro dynamic cancer invasion model, different cancer cell types showed distinct physiological and genetic characteristics of invasiveness. All three types of CAFs differentiated by cancer cell-derived exosomes were enriched in ECM to promote the invasion process. However, invasive melanoma cells were associated with AKT1 signaling related to the migration pathway, squamous cells with the EGFR signaling in response to extracellular cues and breast cancer cells with IL-6 signaling involved in the inflammatory response. In addition, The DE gene levels of ecCAFs showed enhanced angiogenesis and focal adhesion-related signaling pathways compared to cCAFs. Our findings validate application of the 3D microfluidic system that mimics the in vivo cancer microenvironment in invasion research. Future studies will focus on CAF differentiation of diverse cell types and cell-to-cell interactions that contribute to invasion mechanisms. We additionally aim to establish methods for preventing cancer invasion at the molecular level, with a view to ultimately developing therapeutic agents that inhibit CAF differentiation and cancer progression.

## Supplementary Materials

The following are available online at https://www.mdpi.com/1422-0067/21/21/8153/s1.

## References

[1] Friedl P., Wolf K. (2003). Tumour-cell invasion and migration: Diversity and escape mechanisms. Nat. Rev. Cancer.

[2] Steeg P.S. (2016). Targeting metastasis. Nat. Rev. Cancer.

[3] Weidner N., Semple J.P., Welch W.R., Folkman J. (1991). Tumor angiogenesis and metastasis—correlation in invasive breast carcinoma. N. Engl. J. Med..

[4] Hunter K.W., Crawford N.P., Alsarraj J. (2008). Mechanisms of metastasis. Breast Cancer Res..

[5] Joyce J.A., Pollard J.W. (2009). Microenvironmental regulation of metastasis. Nat. Rev. Cancer.

[6] Quail D.F., Joyce J.A. (2013). Microenvironmental regulation of tumor progression and metastasis. Nat. Med..

[7] Zeisberg E.M., Tarnavski O., Zeisberg M., Dorfman A.L., McMullen J.R., Gustafsson E., Chandraker A., Yuan X., Pu W.T., Roberts A.B. (2007). Endothelial-to-mesenchymal transition contributes to cardiac fibrosis. Nat. Med..

[8] Truong D.D., Kratz A., Park J.G., Barrientos E.S., Saini H., Nguyen T., Pockaj B., Mouneimne G., LaBaer J., Nikkhah M. (2019). A human organotypic microfluidic tumor model permits investigation of the interplay between patient-derived fibroblasts and breast cancer cells. Cancer Res..

[9] Nguyen M., De Ninno A., Mencattini A., Mermet-Meillon F., Fornabaio G., Evans S.S., Cossutta M., Khira Y., Han W., Sirven P. (2018). Dissecting effects of anti-cancer drugs and cancer-associated fibroblasts by on-chip reconstitution of immunocompetent tumor microenvironments. Cell Rep..

[10] Yan Y., Wang L.-F., Wang R.-F. (2015). Role of cancer-associated fibroblasts in invasion and metastasis of gastric cancer. World J. Gastroenterol..

[11] Pelon F., Bourachot B., Kieffer Y., Magagna I., Mermet-Meillon F., Bonnet I., Costa A., Givel A.-M., Attieh Y., Barbazan J. (2020). Cancer-associated fibroblast heterogeneity in axillary lymph nodes drives metastases in breast cancer through complementary mechanisms. Nat. commu..

[12] Sun Q., Zhang B., Hu Q., Qin Y., Xu W., Liu W., Yu X., Xu J. (2018). The impact of cancer-associated fibroblasts on major hallmarks of pancreatic cancer. Theranostics.

[13] Prakash J. (2016). Cancer-associated fibroblasts: perspectives in cancer therapy. Trends Cancer.

[14] Erdogan B., Ao M., White L.M., Means A.L., Brewer B.M., Yang L., Washington M.K., Shi C., Franco O.E., Weaver A.M. (2017). Cancer-associated fibroblasts promote directional cancer cell migration by aligning fibronectin. J. Cell Biol..

[15] Saini H., Rahmani K., Veldhuizen J., Zare A., Allam M., Silva C., Kratz A., Truong D., Mouneimne G., LaBaer J. (2020). The role of tumor-stroma interactions on desmoplasia and tumorigenicity within a microengineered 3D platform. Biomaterials.

[16] Clere N., Renault S., Corre I. (2020). Endothelial-to-Mesenchymal Transition in Cancer. Front. Cell Dev. Biol..

[17] Choi K.J., Nam J.-K., Kim J.-H., Choi S.-H., Lee Y.-J. (2020). Endothelial-to-mesenchymal transition in anticancer therapy and normal tissue damage. Exp. Mol. Med..

[18] Jiao K., Zhen J., Wu M., Teng M., Yang K., Zhou Q., Hu C., Zhou M., Li Y., Li Z. (2020). 27-Hydroxycholesterol-induced EndMT acts via STAT3 signaling to promote breast cancer cell migration by altering the tumor microenvironment. Cancer Biol. Med..

[19] Motohara T., Masuda K., Morotti M., Zheng Y., El-Sahhar S., Chong K.Y., Wietek N., Alsaadi A., Karaminejadranjbar M., Hu Z. (2019). An evolving story of the metastatic voyage of ovarian cancer cells: Cellular and molecular orchestration of the adipose-rich metastatic microenvironment. Oncogene.

[20] Yoshida G.J. (2020). Regulation of heterogeneous cancer-associated fibroblasts: The molecular pathology of activated signaling pathways. J. Exp. Clin. Cancer Res..

[21] Piper M., Mueller A.C., Karam S.D. (2020). The interplay between cancer associated fibroblasts and immune cells in the context of radiation therapy. Mol. Carcinog..

[22] Yeon J.H., Jeong H.E., Seo H., Cho S., Kim K., Na D., Chung S., Park J., Choi N., Kang J.Y. (2018). Cancer-derived exosomes trigger endothelial to mesenchymal transition followed by the induction of cancer-associated fibroblasts. Acta Biomater..

[23] Kim H., Lee S., Shin E., Seong K.M., Jin Y.W., Youn H., Youn B. (2020). The Emerging Roles of Exosomes as EMT Regulators in Cancer. Cells.

[24] Bussard K.M., Mutkus L., Stumpf K., Gomez-Manzano C., Marini F.C. (2016). Tumor-associated stromal cells as key contributors to the tumor microenvironment. Breast Cancer Res..

[25] Othman N., Jamal R., Abu N. (2019). Cancer-derived exosomes as effectors of key inflammation-related players. Front. Immunol..

[26] Hu C., Chen M., Jiang R., Guo Y., Wu M., Zhang X. (2018). Exosome-related tumor microenvironment. J. Cancer.

[27] Kikuchi S., Yoshioka Y., Prieto-Vila M., Ochiya T. (2019). Involvement of extracellular vesicles in vascular-related functions in cancer progression and metastasis. Int. J. Mol. Sci..

[28] Conigliaro A., Cicchini C. (2019). Exosome-mediated signaling in epithelial to mesenchymal transition and tumor progression. J. Clin. Med..

[29] Richards K.E., Zeleniak A.E., Fishel M.L., Wu J., Littlepage L.E., Hill R. (2017). Cancer-associated fibroblast exosomes regulate survival and proliferation of pancreatic cancer cells. Oncogene.

[30] Ruivo C.F., Adem B., Silva M., Melo S.A. (2017). The biology of cancer exosomes: Insights and new perspectives. Cancer Res..

[31] Sun W., Luo J.-d., Jiang H., Duan D.D. (2018). Tumor exosomes: A double-edged sword in cancer therapy. Acta Pharmacol. Sin..

[32] Xiao Y., Zhong J., Zhong B., Huang J., Jiang L., Jiang Y., Yuan J., Sun J., Dai L., Yang C. (2020). Exosomes as potential sources of biomarkers in colorectal cancer. Cancer Lett..

[33] Wu Y., Deng W., Klinke II D.J. (2015). Exosomes: Improved methods to characterize their morphology, RNA content, and surface protein biomarkers. Analyst.

[34] Ning X., Zhang H., Wang C., Song X. (2018). Exosomes released by gastric cancer cells induce transition of pericytes into cancer-associated fibroblasts. Med. Sci. Monit..

[35] Erdogan B., Webb D.J. (2017). Cancer-associated fibroblasts modulate growth factor signaling and extracellular matrix remodeling to regulate tumor metastasis. Biochem. Soc. Trans..

[36] Saiki I., Murataxd J., Yoneda J., Kobayashi H., Azuma I. (1994). Influence of fibroblasts on the invasion and migration of highly or weakly metastatic B16 melanoma cells. Int. J. Cancer.

[37] Nicolson G.L., Dulski K., Basson C., Welch D.R. (1985). Preferential organ attachment and invasion in vitro by B16 melanoma cells selected for differing metastatic colonization and invasive properties. Invasion Metastasis.

[38] Didona D., Paolino G., Bottoni U., Cantisani C. (2018). Non melanoma skin cancer pathogenesis overview. Biomedicines.

[39] Apalla Z., Nashan D., Weller R.B., Castellsague X. (2017). Skin cancer: Epidemiology, disease burden, pathophysiology, diagnosis, and therapeutic approaches. Dermatol. Ther..

[40] Martinez J.-C., Otley C.C. (2001). The management of melanoma and nonmelanoma skin cancer: A review for the primary care physician. Mayo Clin. Proc..

[41] Sonnenschein C., Soto A.M. (2015). Cancer metastases: So close and so far. J. Natl. Cancer Inst..

[42] Chen A., Wang L., Liu S., Wang Y., Liu Y., Wang M., Nakshatri H., Li B.-Y., Yokota H. (2018). Attraction and Compaction of Migratory Breast Cancer Cells by Bone Matrix Proteins through Tumor-Osteocyte Interactions. Sci. Rep..

[43] Dasari S., Fang Y., Mitra A.K. (2018). Cancer associated fibroblasts: Naughty neighbors that drive ovarian cancer progression. Cancers.

[44] Wortzel I., Dror S., Kenific C.M., Lyden D. (2019). Exosome-mediated metastasis: Communication from a distance. Dev. Cell.

[45] Cesano A. (2015). nCounter^®^ PanCancer immune profiling panel (NanoString technologies, Inc., Seattle, WA). J. Immunother. Cancer.

[46] Suh S.-S., Yoo J.Y., Cui R., Kaur B., Huebner K., Lee T.-K., Aqeilan R.I., Croce C.M. (2014). FHIT suppresses epithelial-mesenchymal transition (EMT) and metastasis in lung cancer through modulation of microRNAs. PLoS Genet.

[47] Shintani Y., Fujiwara A., Kimura T., Kawamura T., Funaki S., Minami M., Okumura M. (2016). IL-6 secreted from cancer-associated fibroblasts mediates chemoresistance in NSCLC by increasing epithelial-mesenchymal transition signaling. J. Thorac. Oncol..

[48] Goulet C.R., Champagne A., Bernard G., Vandal D., Chabaud S., Pouliot F., Bolduc S. (2019). Cancer-associated fibroblasts induce epithelial–mesenchymal transition of bladder cancer cells through paracrine IL-6 signalling. BMC Cancer.

[49] Stivarou T., Patsavoudi E. (2015). Extracellular molecules involved in cancer cell invasion. Cancers.

[50] Ciavarella S., Laurenzana A., De Summa S., Pilato B., Chilla A., Lacalamita R., Minoia C., Margheri F., Iacobazzi A., Rana A. (2017). u-PAR expression in cancer associated fibroblast: New acquisitions in multiple myeloma progression. BMC Cancer.

[51] Chen X., Song E. (2019). Turning foes to friends: Targeting cancer-associated fibroblasts. Nat. Rev. Drug Discov..

[52] Daley W.P., Peters S.B., Larsen M. (2008). Extracellular matrix dynamics in development and regenerative medicine. J. Cell Sci..

[53] Kumari S., Malla R. (2015). New insight on the role of plasminogen receptor in cancer progression. Cancer Growth Metastasis.

[54] Quemener C., Gabison E.E., Naïmi B., Lescaille G., Bougatef F., Podgorniak M.P., Labarchède G., Lebbé C., Calvo F., Menashi S. (2007). Extracellular matrix metalloproteinase inducer up-regulates the urokinase-type plasminogen activator system promoting tumor cell invasion. Cancer Res..

[55] Langhammer S., Scheerer J. (2017). Breaking the crosstalk of the cellular tumorigenic network: Hypothesis for addressing resistances to targeted therapies in advanced NSCLC. Oncotarget.

[56] Zhang X., Hwang Y.S. (2019). Cancer-associated fibroblast stimulates cancer cell invasion in an interleukin-1 receptor (IL-1R)-dependent manner. Oncol. Lett..

[57] Gallagher S., Tiffen J., Hersey P. (2015). Histone modifications, modifiers and readers in melanoma resistance to targeted and immune therapy. Cancers.

[58] Yoshida G.J., Azuma A., Miura Y., Orimo A. (2019). Activated fibroblast program orchestrates tumor initiation and progression; molecular mechanisms and the associated therapeutic strategies. Int. J. Mol. Sci..

[59] Hirata E., Girotti M.R., Viros A., Hooper S., Spencer-Dene B., Matsuda M., Larkin J., Marais R., Sahai E. (2015). Intravital imaging reveals how BRAF inhibition generates drug-tolerant microenvironments with high integrin β1/FAK signaling. Cancer Cell.

[60] Chan X., Singh A., Osman N., Piva T. (2017). Role played by signalling pathways in overcoming BRAF inhibitor resistance in melanoma. Int. J. Mol. Sci..

[61] Rosell R., Karachaliou N., Morales-Espinosa D., Costa C., Molina M.A., Sansano I., Gasco A., Viteri S., Massuti B., Wei J. (2013). Adaptive resistance to targeted therapies in cancer. Transl. Lung Cancer Res..

[62] Mainiero F., Murgia C., Wary K.K., Curatola A.M., Pepe A., Blumemberg M., Westwick J.K., Der C.J., Giancotti F.G. (1997). The coupling of α6β4 integrin to Ras–MAP kinase pathways mediated by Shc controls keratinocyte proliferation. EMBO J..

[63] Culig Z. (2011). Cytokine disbalance in common human cancers. BBA Mol. Cell. Res..

[64] Sirotković-Skerlev M., Kulić A., Bradić L., Čačev T. (2012). Protumor effects of proinflammatory mediators in breast cancer. Period. Biol..

[65] Benedicto A., Romayor I., Arteta B. (2017). Role of liver ICAM-1 in metastasis. Oncol. Lett..

[66] Gialeli C., Viola M., Barbouri D., Kletsas D., Passi A., Karamanos N. (2014). Dynamic interplay between breast cancer cells and normal endothelium mediates the expression of matrix macromolecules, proteasome activity and functional properties of endothelial cells. Biochim. Biophys. Acta Gen. Subj..

