# Supplementary material for: Cancer-Associated Fibroblasts Differentiated by Exosomes Isolated from Cancer Cells Promote Cancer Cell Invasion
Source: Int J Mol Sci. 2020 Oct 31;21(21):8153. doi: 10.3390/ijms21218153 (PMC7662577; doi:10.3390/ijms21218153)
Supplement: Supplementary file 1 [file ijms-21-08153-s001.pdf]

Supplementary data for

Cancer-associated fibroblasts differentiated by exosomes isolated from cancer cells promote cancer cell invasion

Kimin Kim[1†], Yeh Joo Sohn[1†], Ruri Lee[1], Hye Ju Yoo[1], Ji Yoon Kang[2,3], Nakwon Choi[2,3], Dokyun Na[4*], Ju Hun Yeon[1*]

[1]Department of Integrative Biosciences, University of Brain Education (UBE), Cheonan 31228, Republic of Korea

[2]Center for BioMicrosystems, Brain Science Institute, Korea Institute of Science and Technology (KIST), 02792 Seoul, Republic of Korea

[3]Division of Bio-Medical Science & Technology (Biomedical Engineering), KIST school, Korea University of Science and Technology (UST), 02792 Seoul, Republic of Korea

[4]School of Biomedical Engineering, Chung-Ang University, 06974 Seoul, Republic of Korea

*Co-corresponding authors.

E-mail address: jhyeon@ube.ac.kr

Tel.: +82-41-529-2621

Fax.: +82-41-529-2674

Dokyun Na

E-mail address: blisszen@cau.ac.kr

Tel: +82-2-820-5690

Fax: +82-2-814-2651

Keywords: cancer-associated fibroblasts, cancer cells-derived exosomes, invasive cancer cells, cancer cell invasion, 3D microfluidics

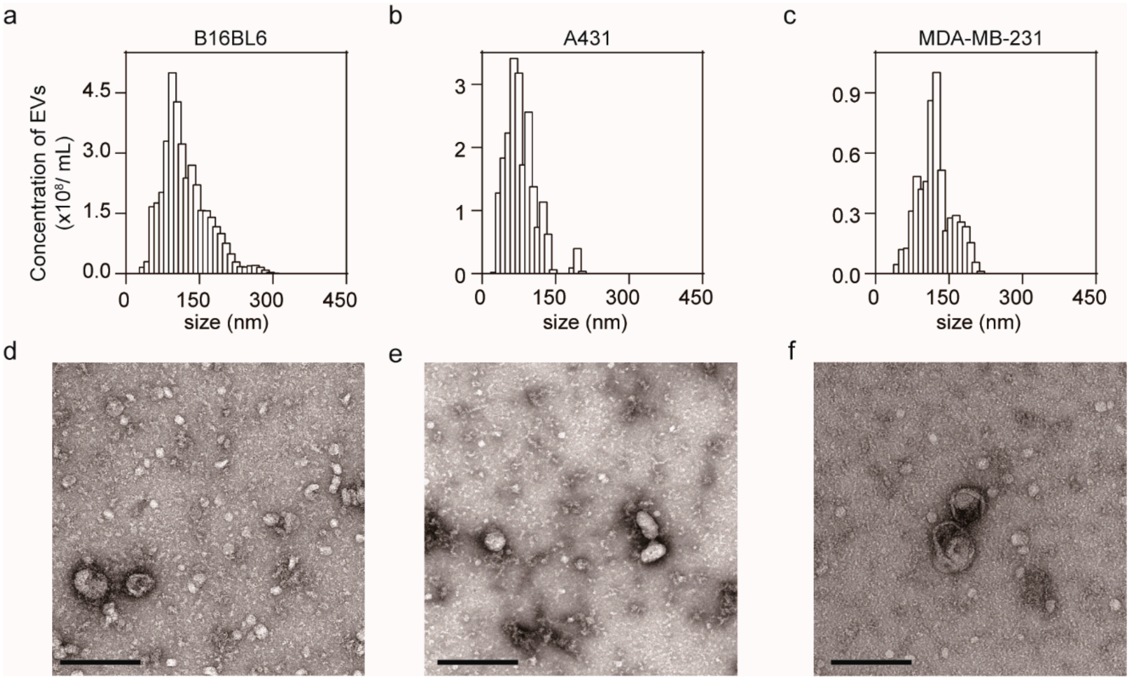

Supplementary Figure 1. Characterization of isolated exosomes from the three cancer cell lines. (a-c) Nanoparticle tracking analysis (NTA) measurements of concentration and size distribution of exosomes from B16BL6, A431, and MDA-MB-231 cells. (d-f) Transmission electron microscopy (TEM) images of exosomes from B16BL6, A431 and MDA-MB-231 cells (Scale bar: 200 nm).

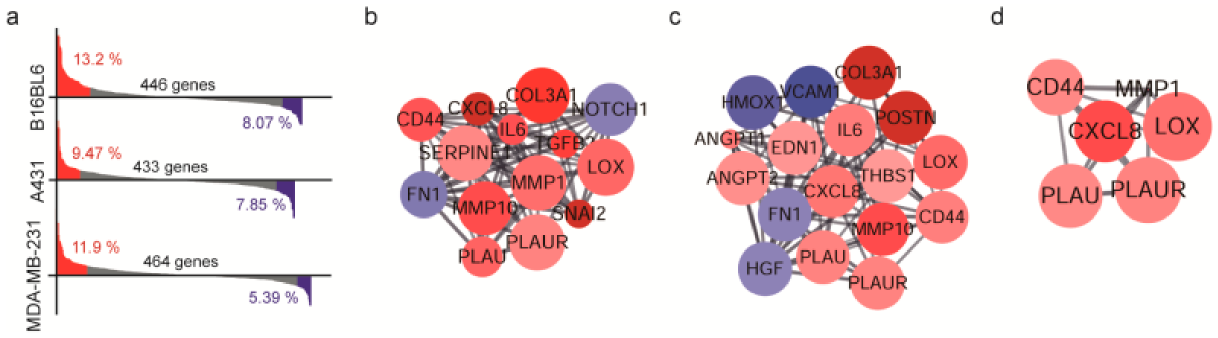

Supplementary Figure 2. Identification of genes and functions associated with CAFs induced by cancer cells-derived exosomes (eCAFs) derived from cancer cells, respectively.

(a) Expression levels of mRNAs in eCAFs differentiation-triggered by the exosomes extracted from B16BL6, A431, MDA-MB-231 cells. (b-d) Top module of protein-protein interaction (PPI) network for densely connected nodes. Red DEs with $\log_2$fold change >1; B16BL6, A431, MDA-MB-231 cells. Blue DEs with $\log_2$fold change <-1. The larger node size, the more significant p-values.

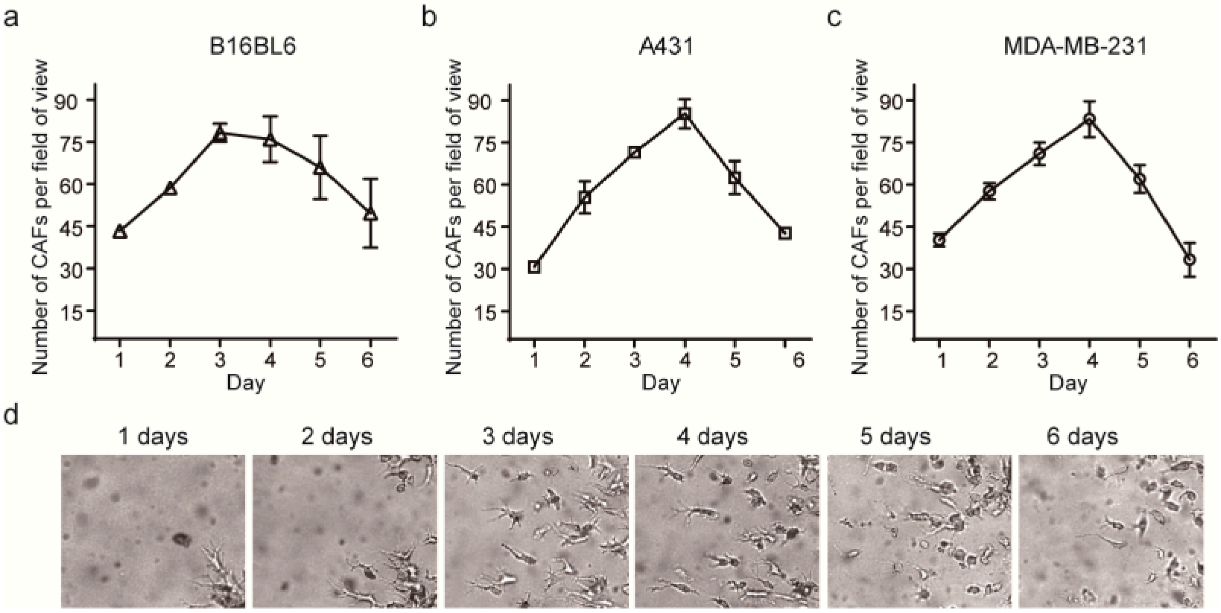

Supplementary Figure 3. Cancer-associated fibroblasts formation in the microfluidic device.
(a) B16BL6, (b) A431, (c) MDA-MB-231 cells-derived exosomes increased the number of CAFs, which was highest after 3-4 days, and then gradually decreased. (d) Growth image of CAFs induced by B16BL6 cells-derived exosomes (50 μg/mL) for 6 days.
